# Study on the Mechanical Properties of Bionic Protection and Self-Recovery Structures

**DOI:** 10.3390/ma13020389

**Published:** 2020-01-15

**Authors:** Xue Guo, Xinju Dong, Zhenglei Yu, Zhihui Zhang, Xinyu Xie, Xiebin Wang, Renlong Xin, Wei Yan

**Affiliations:** 1 Key Lab of Bionic Engineering, Ministry of Education, Jilin University, Changchun 130000, China; guoxue1817@mails.jlu.edu.cn (X.G.); dongxj1817@mails.jlu.edu.cn (X.D.); zhzh@jlu.edu.cn (Z.Z.);; 2 School of Materials Science and Engineering, Shandong University, Jinan 250000, China; wangxiebin@sdu.edu.cn; 3 Beijing Huashengyang Hi-Tech Co., Ltd., Beijing 100000, China; yanw@hsy-tech.com

**Keywords:** bionic protective structure, NiTi alloy, compression characteristics, shape memory properties, numerical simulation

## Abstract

A novel protective structure, based on shrimp chela structure and the shape of odontodactylus scyllarus, has been shown to improve impact resistance and energy absorption. A finite element model of NiTi alloy with shape memory was constructed based on the basic principles of structural bionics. The protective structure utilizes NiTi alloy as the matrix, a material with many advantages including excellent compression energy absorption, reusability after unloading, and long life. The mechanical properties of the single-layer model were obtained by static crushing experiments and numerical simulations. Building upon the idea of the monolayer bionic structure design, a two-layer structure is also conceived. Both single-layer and double-layer structures have excellent compression energy absorption and self-recovery capabilities. Compared with the single-layer structure, the double-layer structure showed larger compression deformation and exhibited better energy absorption capacity. These results have important academic and practical significance for improving the impact resistance of protective armor. Our study makes it possible to repair automatic rebounds under the action of pressure load and improves the endurance and material utilization rate of other protective structures.

## 1. Introduction

The variety and extent of mechanical damage caused by collision emphasizes the need to develop improved armor protection materials with optimized or upgraded design structures. Presently, commonly used protective materials include metal materials, ceramic materials, composite materials, and so on. The structural design mainly includes honeycomb structures, sandwich structures, and metal seal structures. Each material and structure has its own advantages and disadvantages. Thus, improving and upgrading materials and structures are the directions for the development of protective armors in the future.

With the development of modern science and engineering technology, new composite materials with excellent mechanical properties such as light weight, high strength, and impact resistance have been invented and have wide application prospects in aerospace, automotive, ships, machinery manufacturing, and many other fields. These composites have high compressive strength and impact toughness [1,2,3,4]. Zhou [1] investigated the penetration of a composite armor of 30CrMnMo (a medium carbon low alloy steel) and ultra-high molecular weight polyethylene (PE) by a high-speed fragment via experiments and simulations. The results show that PE laminates absorb energy through the continuous stable failure of PE fibers at the beginning of penetration, and absorb energy through deformation until complete penetration. It was concluded that the energy absorbed by laminates accounts for 68% of the total energy absorption. Dov [2] studied the quasi-static and dynamic damage mechanisms of alumina tiles under a variety of boundary conditions. Impact damage of laminated B4C ceramic samples was also examined using four types of aluminum sheets [3]. Finally, a ceramic-metal composite structure was established by using TC4 frame and ceramic prism structure, and its anti-penetration protection performance were inspected by experiments and numerical simulations [4].

Due to the optimization process in evolution, the structure and function of biological materials have provided concepts to solve many modern engineering problems. These bionic investigations have promoted the development of science and technology. For example, shellfish and crustaceans in nature not only have the characteristics of light weight, high hardness, and high strength but also have good impact resistance, which have greatly inspired the biomimetic research in the field. Biomimetic coupled layered B4C/5083AL composites have been fabricated by hot press sintering according to the microstructural characteristics of the shell [5]. The internal structure of crustaceans was found to be arranged in a spiral structure of fiber layers, a structure that can resist shock and promote energy absorption [6]. Many natural biomaterials contain properties of energy absorption, which has provided a significant reference for solving the problem of buffering performance and collision protection. Enhanced energy absorption capability was found in many bio-inspired structures. In order to improve the energy absorption characteristics of thin-wall tubular structures, Hu [7], inspired by the microstructure of bamboo vascular bundles, simulated the natural geometric shape and developed a bionic honeycomb tubular nesting structure. The preliminary results showed that the honeycomb tubular nesting structure had a good energy absorption performance. Compared with bamboo, wood has a stronger anisotropy in its microstructure. Through the combination of geometric modeling, finite element analysis, and off-axis compression tests, Arnaud Marmier [8] demonstrated that the negative Poisson’s ratio of wood has unusual elastic properties.

In the long process of biological evolution, all the creatures of nature have evolved some protection mechanism to adapt to the harsh natural environment. For example, during the long natural evolution process, when prawns were hunting, they would attack animals using their appendages, and so their appendages have excellent impact resistance [9,10,11]. The biological morphology of the mantis shrimp has been imitated and extracted, and the bionic morphology with the same excellent impact resistance has been copied. A hammer can easily break open the shell of crustaceans, shellfish, snails, and other animals. Yet, on the other hand, the shrimp can withstand the impact of more than 700 N. When the shrimp structure is impacted and the load comes into contact with the prey, the load is transferred step by step to achieve a cushioning effect on the energy absorption [12,13]. The sandwich structure can withstand load and resist shock waves better, exhibiting excellent plastic deformation property and energy absorption ability. A 36-caliber core bomb was used to evaluate the protective properties of three different composites at a speed of 700 m/s. It was concluded that plywood used between aramid and carbon-fiber reinforced aluminum honeycomb sandwich board was a reliable and cheap composite protective layer [14]. The dynamic response of honeycomb sandwich beams with negative Poisson’s ratio under local impact has also been studied. It was found that the honeycomb core had local expansion and expansion deformation capacities [15].

As a kind of intelligent material, shape memory alloys (SMA) are widely used in mechanical and automotive industries because of their eponymous shape memory effect. In 1959, William Buehler found the NiTi alloy [16] and in 1963, Buehler found its memory effect [17]. SMAs can undergo phase transition and lattice shear under tensile or compressive loading and absorb a large amount of mechanical energy. In the process of unloading, the material can bounce back to its original state quickly, and undergo inverse phase transition, accompanied by the outward diffusion of a large amount of energy in the form of heat. Memory alloys are materials that have the ability to retain their previous shape when subjected to certain compressive or tensile strains. Thus, they, especially NiTi SMAs, have a wide applications in aerospace, automotive, smart materials, acoustic applications, actuators biomedicine and many other fields [18,19,20,21,22]. Based on the energy absorption characteristics of NiTi alloy and its shape memory characteristics, a protection structure of NiTi alloy was constructed. It can absorb and consume impact energy, and improve the impact resistance and durability of protective structures.

Recently, plus its impact resistance and compression resilience. The stress and strain curves w, domestic and international scholars in the field of armor protection technology have focused research on material modification and structure design. However, the self-repairing property of protective armors has rarely been investigated. Here, we propose a complete design of the bionic protection self-repair structure based on the biological model. NiTi SMA was used to build this bionic protection self-repair model because of its excellent performance. Second, a single-layer three-dimensional finite element model was established. The stress-strain relationship under compression and unloading conditions was compared and analyzed through the quasi-static collapse test, and the bearing capacity and recovery performance were also studied. The accuracy of simulation analysis was verified by comparing to empirical tests. Third, the single-layer model was upgraded to a double-layer model, and a finite element model and a real model of the double-layer structure were also established. Numerical simulations and experiments were used to examine the buffering and absorbing ability of the double-layer structure are composed under the same loading and unloading conditions, which verify the accuracy of the static test results. Fourth, we present a new material technology for armor protection, in which the material and structure are modified and upgraded to achieve self-recovery after removing the pressure load. Compared with other protective armors, the bionic protective self-repairing model described in this study solved the prototyping issues after removing the pressure load and also further enhanced the mechanical properties of armor.

## 2. Bionic Design

### 2.1. Appendages of Mantis Shrimps

**Figure 1:**
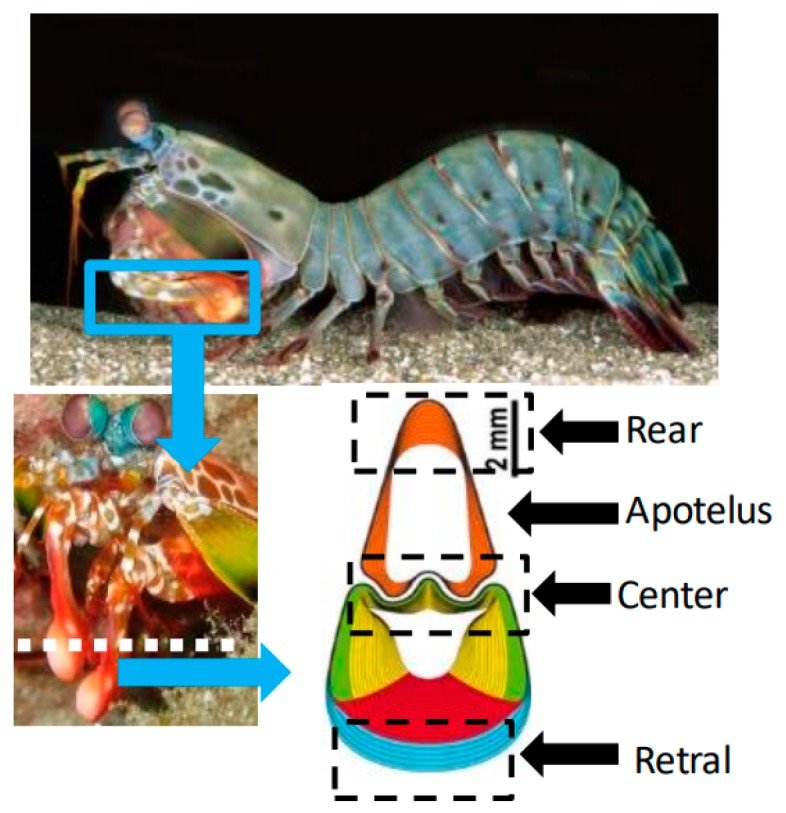
Structure of shrimp chela [11].

The appendages of mantis shrimps have excellent impact resistance. The short limbs of finch tailed mantis shrimps can withstand thousands of blows. The geometry and impact resistance mechanism of the appendage feet of sparrow mantis shrimps were studied here. As shown in Figure 1, the shape of the surface follows rules.

The cavity makes the structure easy to deform on both sides when subjected to external load, increasing the energy absorption capacity of deformation. The findings that shrimp can absorb energy by buffering step by step inspired our design of the double-layer bionic protective structure. The cross section of the mantis shrimp is fusiform, which is composed of two parts—the front toe and the knuckle, both of which contain cavities in the middle. The shrimp shell can withstand high strength impact, and the structure buckles against deformation to absorb impact energy, showing excellent high strength, impact resistance, and other characteristics.

In this paper, a simplified bionic protection model, for easy to prepare, was established by using the shrimp shell, which has good impact resistance, as the biological model.

### 2.2. Material and Fabrication of Bionic Protective Self-Repairing Model Specimen

As a kind of intelligent materials, SMAs are widely used in many engineering fields. NiTi Alloy, a kind of SMAs, with good shape memory characteristics, has many advantages including large compression deformation, high recovery performance, good processing performance, long repeated service life, and low price. Under the action of external stress, the alloy is compressed, distorted, and absorbs energy and then carries out external stimulation to gradually restore the original shape. At the same time, SMAs have a good fatigue resistance and a long service life under cyclic loading and repeated compression. Because of the excellent mechanical property of NiTi alloys, the treated NiTi alloy strips were used as the matrix material in this bionic protection model. Therefore, resulting structure not only exhibits the characteristics of compression, buffer and energy absorption, but also has the excellent recoverability and reusability after compression.

### 2.3. Single-Layer Structure

#### 2.3.1. Numerical Simulation

Finite element analysis is one of the most widely used numerical analysis methods. With the improvement and development of science and technology, the finite cloud method is being continuously improved, and the error between the results obtained by finite element analysis and the actual results has become smaller and smaller. In order to understand the process of the quasi-static test, reduce the cost and time of the real-time test, and carry on further research, a quasi-static crushing experiment simulation was carried out using LS-DYNA (R7.1, Ansys, PA, America).

**Figure 2:**
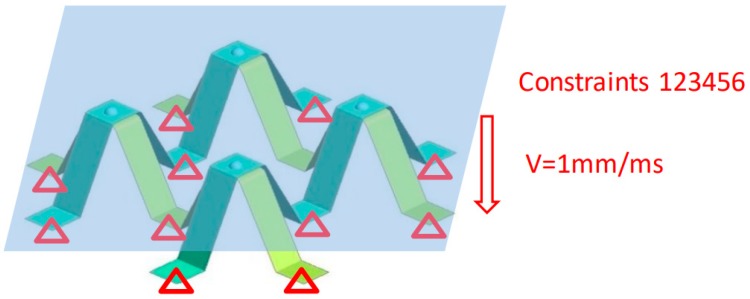
The quasi-static collapse simulation model of memory alloy combination single layer module.

Based on the structure of buffer energy absorption, a bionic protective self-repair model was established by using the grid structure design technology and the lightweight mixed material application technology. The model relies on self-flexion to obtain buffer energy absorption and pressure protection ability. In the process of deformation recovery, NiTi SMA can convert external mechanical energy into internal energy, and then release it in the form of heat, so as to realize the self-repair characteristics of the model. As attributed to NiTi alloy’s shape memory properties and the appendages of mantis shrimps, the bionic protective self-repairing model has superior mechanical properties including compressive strength and self-restoring performance, as compared to existing armor protector. Finite element (FE) model was developed using LS-DYNA package to simulate the behavior of the specimens under quasi–static compression. FE model was simulated by 16 fully integrated shell elements. The current FE model was established using element size of 0.5 mm. The full degree of freedom constraint was applied to the bottom of the model, as shown in Figure 2. The rigid wall had a velocity of 1 mm/ms at the top of the model.

#### 2.3.2. Fabrication of Bionic Protective Self-Repairing Model Specimen

**Figure 3:**
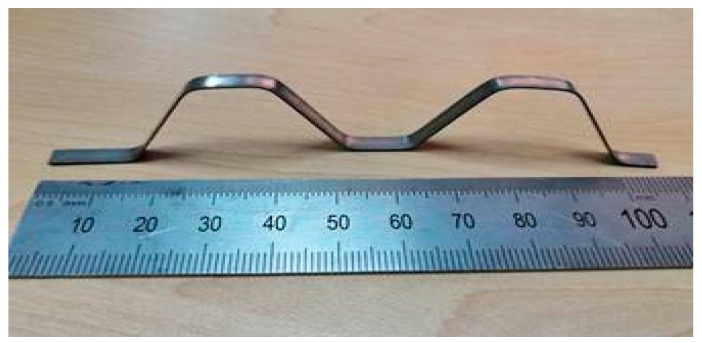
Monomer of NiTi SMA bracket.

**Figure 4:**
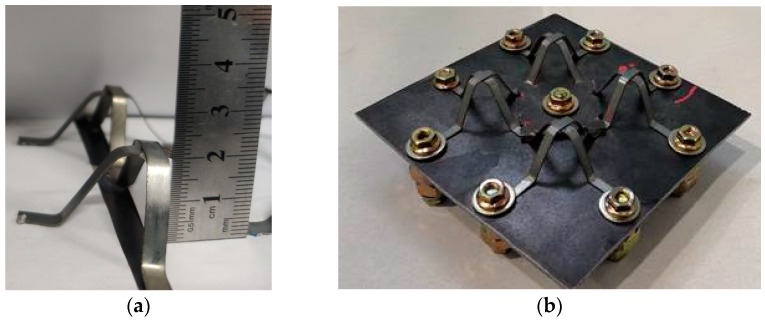
(**a**) The height of Monomer of NiTi SMA bracket, (**b**) NiTi SMA bracket combination module.

NiTi alloy strips were used as the structural material to prepare bionic models, and prepared by cutting, heat treatment, shape training and other methods as shown in Figure 3. Since the shape of appendages of mantis shrimps is relatively complex, we considered universality and took advantage of symmetry to simplify the shape structures. We used NiTi alloy as a matrix material because it has good chemical stability, high elastic modulus, high corrosion resistance and welding properties. The structure consisting of four NiTi article alloys can provide self-recovery properties, which are inaccessible in previous reports. A model sample welded with NiTi shape memory alloys was used as space truss support; the bionic protective model dimensions were 100 mm (width) × 100 mm (length). The thickness of the monomer is 0.5 mm, the thickness of the monomer is 5 mm, and the total height of the specimen is 20 mm, as depicted in Figure 4.

#### 2.3.3. Experimental Method

**Figure 5:**
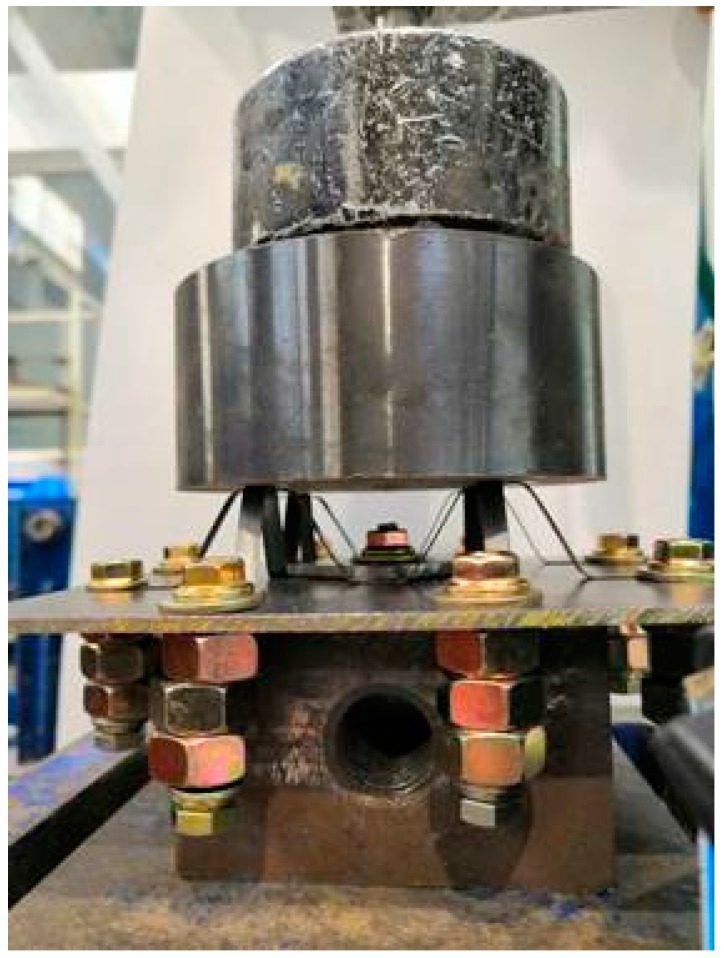
Device diagram of experiment.

The model sample was fixed in the center of the table panel, and the compression characteristics of the model sample were tested by a stiffness measuring device on a load test bench. As shown in Figure 5, the test device was composed of a fixed steel table, plus the force sensor and displacement sensor. The rigid loading plate pressed down onto the tested sample in the direction of gravity to ensure a uniform distribution of impact force. The impact force and strain time-history curve were recorded during the course of the experiment. The time curve of the impact force was measured by a force sensor between the heavy load and sample. As in the simulation procedure, an initial velocity of 1 mm/ms was assigned to the top rigid wall. The laboratory temperature was 24 °C, and the humidity was 40%.

#### 2.3.4. Boundary Conditions

As shown in Table 1, the mechanical properties of NiTi alloys were measured by a quasi-static compression experiment.

## 3. Quasi-Static Compression Experiment Result and Analysis

**Figure 6:**
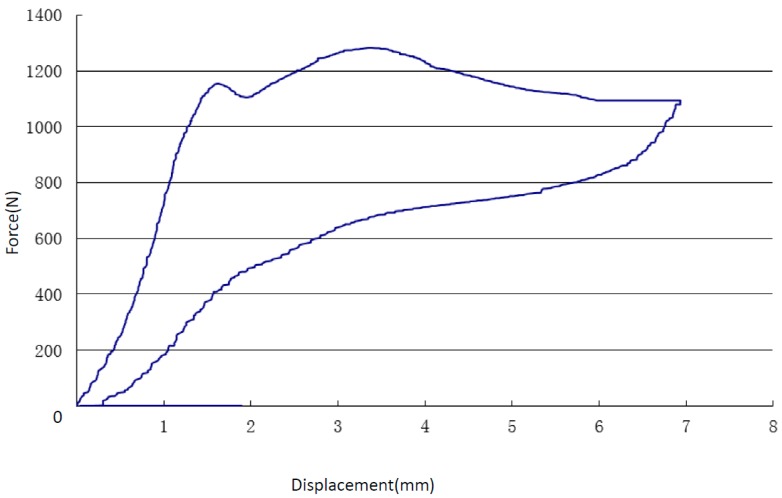
Force–displacement graph hydrostatic experiment.

In the early stage of the rigid loading plate pressing down, due to the strength of NiTi SMA, the module hardly exhibits obvious plastic deformation, and the bearing capacity of the model increased significantly. When the press displacement increased, the bending deformation occurred gradually, and the deformations were concentrated in the position of the lateral diagonal brace. It is unlikely the model provided enough bearing capacity, as the load curve had no obvious rising trend. Figure 6 shows the force–displacement graph in quasi-static experiment. During the pressure process, the bionic model was maximally deformed under the compression of the load.

**Figure 7:**
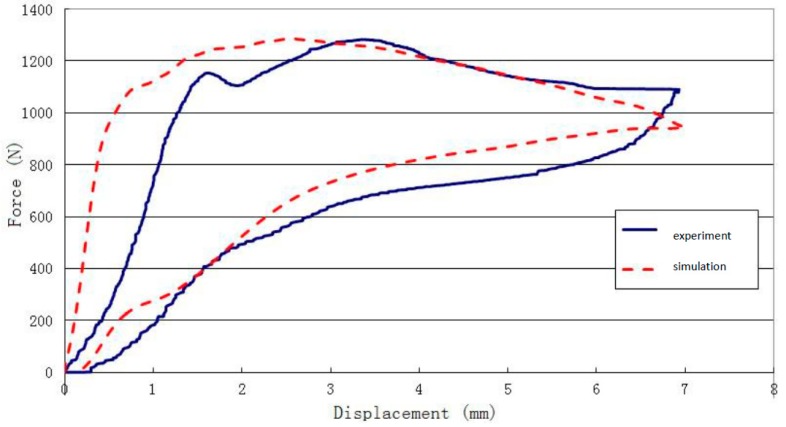
Comparison of the force–displacement curves of the quasi-static experiment and numerical analysis.

As can be seen in Figure 6, strain decreased with the increase of stress, while buckling deformation held relatively stable. After hitting the compression breaking point, the model reached its maximum deformation step by step. The maximum compression stress was 1281 N, and the overall pressing distance was 7 mm, suggesting a good degree of protection power in the vertical direction. In the unloading stage, the NiTi SMA bracket combination module has evident recovery deformation. The recovery force gradually decreased. Therefore, the bionic structure can transform the kinetic energy of the top rigid wall to the NiTi alloy internal energy. After completely unloading, the model was sat for a period of time at room temperature. As can be seen in Figure 7, all of the structure remained intact, and the recovery rate was above 99%. Contrasted to the load curve, the rebound curve was relatively smooth.

Under compressive loading, the NiTi alloy bionic structure buffered the energy of the impact load via both deformation and absorption of the energy carried by the pressing process. The structure restored to its initial shape and the properties remained invariable after the external pressure were removed.

**Figure 8:**
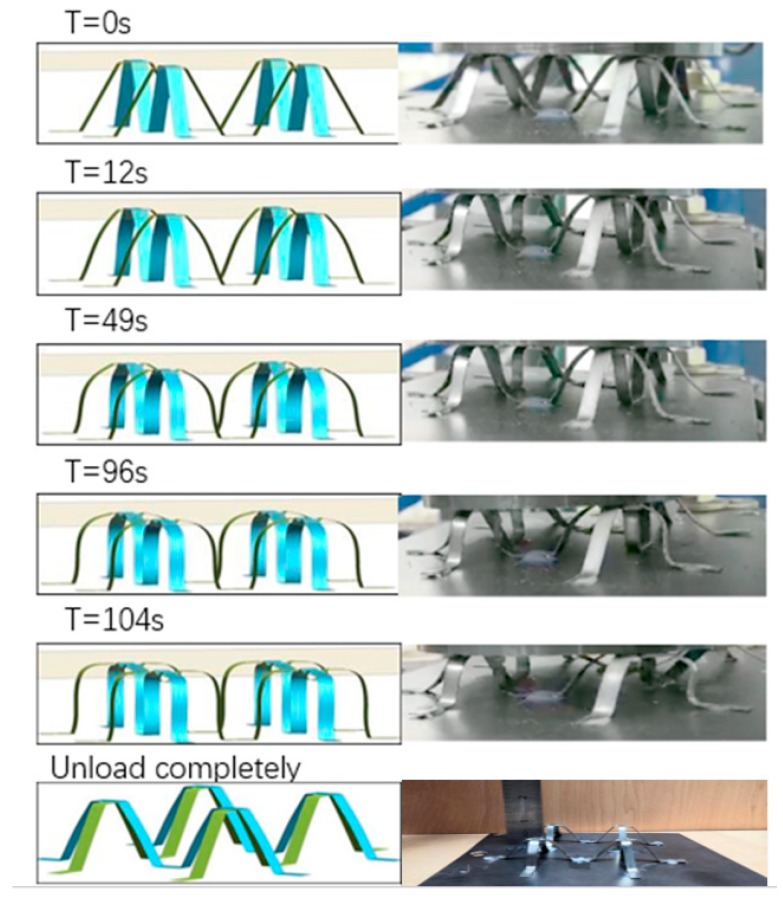
Comparison of simulation and experiment loading procedure of NiTi memory alloy assembly module.

In order to verify the compression limit and resilience, the crushing process of the model sample was simulated. Figure 8 shows a photo of the bionic structure impacted at a velocity of 1 mm/ms. At specific time points, the quasi-static crushing deformation of NiTi alloy model structure and the finite element model was shown. From this figure, the simulation model reached the maximum shape change gradually during the process of applying load pressure. As compared with the empirical test, the results show that the simulation was in good agreement with the deformation state of the test at different moments-the location of plastic hinge appeared similar. The simulation results show that the structure returns to the initial state after unloading, with only 0.16 mm residual deformation; this result is consistent with the overall experimental data of the module. As discussed above, the bionic structure absorbed energy only by small amounts in the first 12 s.

The consistence between experimental and simulation results are shown in Figure 8. Compared with the experimental results, the slope of the simulated compression curve was significantly higher than that of the crushing experiment when the model reached the compression limit. The initial peak load values of the two curves were similar, with a slight difference in the timeframe when the peak was reached. In addition, during the whole process of compression, the physical model showed damage, leading to some fluctuation of the downforce curve. The average crushing load of numerical simulation and test remained basically the same. In all, the results suggest that the bionic structure we designed can improve the energy absorption performance against impact.

## 4. Double-Layer Structures

### 4.1. Simulation Analysis

The double-layer structure was fabricated in the same way as the single-layer structure. According to the material parameters of NiTi SMA, the static pressure test and simulation results of the single layer module were analyzed, and a structural scheme of NiTi SMA double layer composite module was designed. A finite element model of the proposed double-layer structure was also made. This provided a reference for transitioning the design of our bionic protective structure from a single-layer structure to a multi-layer structure.

**Figure 9:**
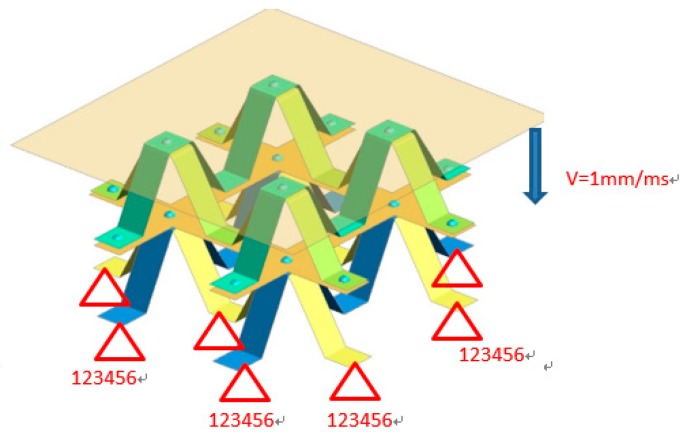
The quasi-static collapse simulation model of memory alloy combination double layer module.

**Figure 10:**
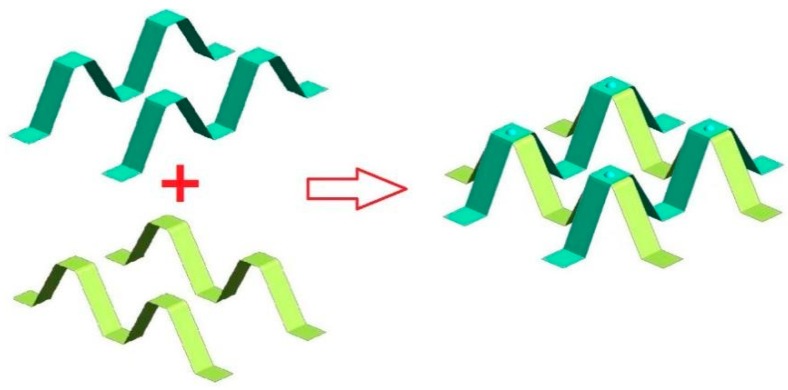
Single layer “M support” welding module.

**Figure 11:**
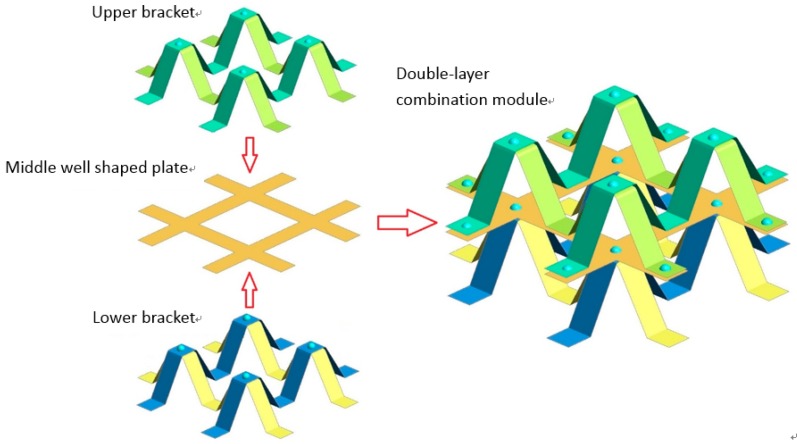
Schematic diagram of interlayer relationship of double-layer composite module.

The bionic protection structure model was established by CATIA three-dimensional software, and geometric modeling was carried out. Solid elements were selected in the analysis. For the reliability of results, the finite element models were meshed by pre-processing software HyperMesh (14.0, Altair, MI, America) and the bionic structure were meshed into 86,560 elements with an element size of 0.5 mm. In order to improve the stability of the bionic protective self-restoring structure, the bottom nodes of the brackets in the composite module were fully constrained. Taking the actual sample test process as the standard, the compression load was removed after pressing 18 mm until the rigid wall was completely separated from the sample. Using the dynamic finite element software LS-DYNA (R7.1, Ansys, PA, America), the deformation process of the rigid wall in loading and unloading stages was analyzed and the rigid wall contact curve versus time was outputted, as can be seen in Figure 9. Bionic protective self-repairing models establish the connection between bionic design and bionic fabrication. Based on our former trials and experiments, the material properties of the double-layer NiTi alloy model were obtained. The process of model of memory alloy combination double layer module were shown in Figure 10 and Figure 11.

The full integral shell element No. 16 was used for the model. The shell element size is 0.5 mm, in which the thickness of each “M-shaped bracket” and “well shaped plate” is 0.5 mm, the width is 10 mm, and the height of a single “M-shaped bracket” is about 20 mm. The solder joint unit between the layers is considered a solid element. The characteristic curve of NiTi SMA material is shown in Table 1, and the related simulation parameters are defined in the MAT_SHAPE_MEMORY keyword. To obtain reliable solutions for further studies of various test conditions, the computer aided simulations were further analyzed. To make useful correlations, the computer simulations were verified with test results.

**Figure 12:**
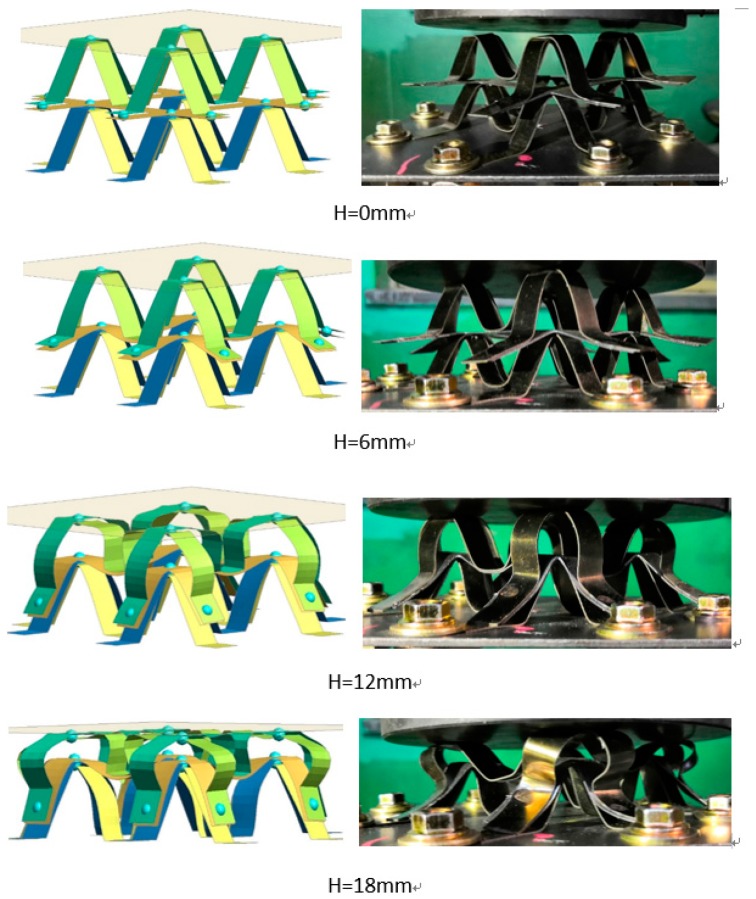
Quasi-static crushing simulation of NiTi memory alloy modular.

In order to further understand the compression process and experimental results of the double layer bionic structure, a simulation was conducted with the models established above. The consistence between experimental and simulation results are shown in Figure 12.

When the rigid loading plate was pressed down 6 mm, the overlap area between the middle “well-shaped plate” and the top of the lower “M-shaped bracket” gradually deformed, and the overlap area between the upper “M-shaped bracket” edge and the middle “well-shaped plate” exhibited a downward bending trend; when the downward pressure was increased to 12 mm, the middle “well-shaped plate” sustained the load transmitted by the upper “M-shaped bracket”. When the rigid loading plate was pressed down to 18 mm, the upper “M-shaped support” appeared unstable and collapses completely, and an overlap area between the edge and the middle “well-shaped plate” appeared.

The oblique supporting column of the lower “M-type bracket” is extruded, which results in obvious deformation of the lower bracket. Considering that the edge structure of the upper “M-type bracket” is contacted with the fixed bottom plate and the restraint nut, the loading was stopped after the pressure reached 18 mm. To summarize, the deformation order of this structure is basically sequential from top to bottom. Thus, to enhance the bearing capacity of the whole structure or to control the deformation order, the strength of the upper support should be properly optimized.

The quasi-static crushing simulation analysis of the two-layer module was carried out to simulate the whole deformation process of the module in the loading stage, which provides a basic model for the multi-layer and multi-parameter optimization of the subsequent NiTi SMA module.

### 4.2. Experimental Results

**Figure 13:**
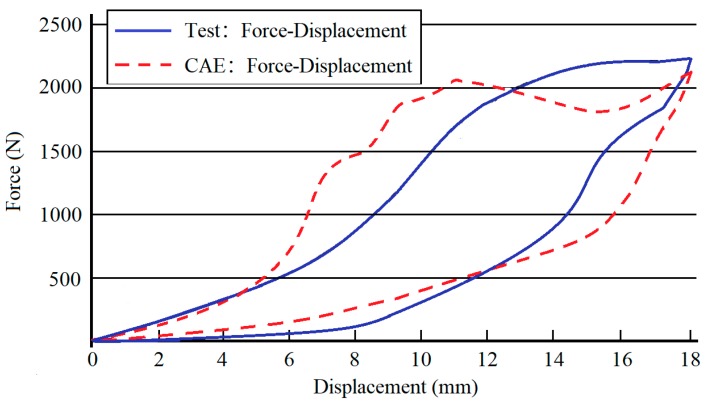
Comparison diagram of load-displacement curve between simulation and test.

**Figure 14:**
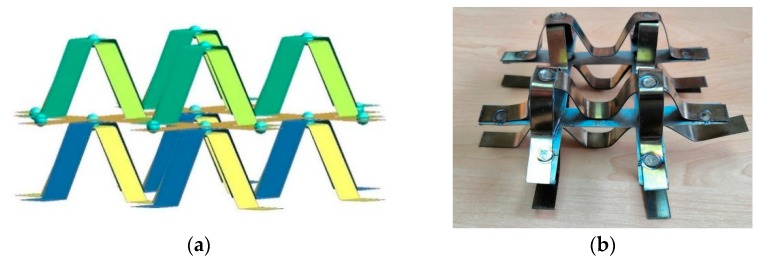
(**a**) After the simulation is completely unloaded, (**b**) Sample of double layer composite module of NiTi SMA.

Figure 13 compares the load-carrying capacity and displacement curves of the simulation analysis and experimental test, including all loading and unloading conditions. It can be seen that the peak values of simulation analysis and empirical test during the loading process are basically the same, and the fluctuation trend is similar. The final state after unloading is also basically the same, as can be seen in Figure 14. Considering slight size differences of “M-type bracket” in the trial production process and the warping deformation of some brackets in the process of layer-by-layer welding, the final test state and simulation conditions may be different. Therefore, other simulation analysis technology can be used to better simulate the deformation process of the NiTi memory alloy double-layer composite module in the process of quasi-static crushing and unloading and rebound and predict its carrying capacity.

Based on the material parameters of NiTi SMA, as well as the static pressure test and simulation analysis results of the single layer module, the structure scheme of a NiTi SMA double layer composite module was designed, and a physical sample was produced. The simulation analysis model of the double-layer composite module under a quasi-static crushing condition was established. The deformation characteristics and bearing capacity curves of double-layer composite module during loading and unloading of simulation analysis and test were compared. We also demonstrated that the simulation analysis can accurately model the overall deformation history and bearing characteristics of the NiTi SMA double-layer composite module. This study contributes to our understanding of the deformation mode of each layer and the law of strength distribution between layers are grasped by this study and better understood, providing an important basis for the optimal design of each layer and the deformation control of the whole module, and presenting an important piece of evidence for expanding the applications and performance design of a multi-layer NiTi alloy.

## 5. Conclusions

In this paper, a bionic protective self-repairing system using NiTi alloy was explored, as inspired by the excellent impact energy absorption of mantis shrimp appendages. We therefore draw the following conclusions:

1. Praying shrimp appendages have excellent impact resistance, and their impact area is hemispherical. This morphology conforms to the principles of structural mechanics and can assist in dispersing the forces they bear.
2. NiTi memory alloys have many advantages including large compression deformation, high recovery performance, good processing performance, long repeated service life, and low price. A protective structure made of NiTi alloy as the matrix material has excellent energy buffering and absorbing ability and the recoverability after compression deformation.
3. Through systematic experiment and simulation analysis, the compression deformation and springback recovery of a single-layer structure under the same working conditions were observed, and the energy absorption characteristics and self-recovery ability of the structure were analyzed. The compression experiments performed on the single-layer structure revealed the mechanical properties of the structure. A numerical method was used to simulate the loading and unloading process of the bionic protection model, and the relative error between the numerical simulation result and the experimental result was found to be no more than 10%, thus verifying the accuracy of the quasi-static crushing experiment and simulation analysis. Experiments and simulation analysis further verify the impact energy absorption and self-recovery characteristics of the single bionic protective self-restoring structure, which can be used to guide the future design of protective armors.
4. The finite element model of a double layer structure was established on the basis of the existing single layer bionic structure and an analysis of compression deformation was carried out by LS-DYNA. Compared with single-layer structure, the double-layer structure was able to resist impact load for a longer time. In the double-layer structure, the intermediate support plate also acts as a stabilizer between the adjacent layers of the multi-layer structure.
5. In the process of loading and unloading, the double model shows very high compression and rebound capability and has strong protection and self-repair characteristics. This provides an accurate compression limit and rebound ratio for the self-restoring structure of the simulated protection material that this paper designed. The bionic protective self-repairing structure is shown to increase the compressive strength and reduce impact damage. At the same time, after removing external pressure, the protective structure can be restored to its original state, which provides critical evidence for the self-restoring technology of sustained protection after any initial perturbation. However, the recovery properties obtained reproducible over a single cycle, are not always recovered after a certain number of cycles. It is well known that SMA materials suffer from unrecoverable effects after a number of cycles due to fatigue.

## Figures and Tables

**Table 1:** Physical properties of NiTi alloys.

Elastic Modulus	Poisson Ratio	Density	Yield Strength	Ultimate Tensile Strength	Total Elongation
~83 GPa (in B2 structured austenite phase)	0.33	6.45 g/cm^3^	195–690 MPa (depending on testing temperatures)	~900 MPa	10–12% (work hardened)
~28–41 GPa (in B19’ structured martensite phase)	–	–	70–140 MPa (depending on testing temperatures)	~2000 MPa (work hardened)	25–60% (fully annealed)
